# Four-branched graft inversion technique for the distal anastomosis in acute aortic dissection

**DOI:** 10.1186/s13019-021-01660-2

**Published:** 2021-10-30

**Authors:** Yu Zou, Peng Teng, Liang Ma

**Affiliations:** Department of Cardiovascular Surgery, The First Affiliated Hospital of Medical College, Zhejiang University, 79 Qing Chun road, Hang Zhou, 310003 Zhejiang Province China

**Keywords:** Aortic arch, Aortic dissection, Bleeding, Frozen elephant trunk, Distal anastomosis

## Abstract

**Background:**

Distal anastomosis bleeding is an issue during total arch replacement with the frozen elephant trunk technique. We used the 4-branched graft inversion technique for the distal anastomosis in acute aortic dissection. The aim was to evaluate the feasibility and benefits of the technique used during the frozen elephant trunk procedure for acute aortic dissection.

**Methods:**

From January 2017 to July 2019, 109 patients underwent total arch replacement for type A acute aortic dissections. Patients were divided according to the technique used for the distal anastomosis as follows: group G (n = 57; 4-branched graft inversion technique) and group C (n = 52; conventional method with Teflon felt). The postoperative variables were analysed.

**Results:**

The hospital mortality rate was 9.2% (10/109). The mean cardiopulmonary bypass, cardiac arrest, and circulatory arrest times were 234.95 ± 71.88 min, 168.25 ± 61.33 min, and 39.19 ± 9.45 min, respectively. The circulatory arrest and cardiac arrest times were shorter in the graft inversion group than in the conventional group (36.46 ± 7.88 min vs. 42.19 ± 10.17 min, *P* = 0.001 and 156.21 ± 55.99 min vs. 181.44 ± 64.68 min, *P* = 0.031, respectively). There were 7 cases of stroke (6.4%) and 5 cases of paraplegia (4.6%). Additionally, 13 patients (11.9%) required temporary continuous renal replacement therapy. Respiratory failure occurred in 19 patients (17.4%). There were no significant differences in postoperative complications between the two groups.

**Conclusions:**

The 4-branched graft inversion technique provides effective and confirmed haemostasis during total aortic arch replacement using the frozen elephant trunk procedure.

## Background

Acute type A aortic dissection is a surgical emergency with a high mortality rate [1]. The prognosis of total aortic replacement for acute type A aortic dissection is unsatisfactory [2], and bleeding is one of the main reasons. Owing to limited surgical exposure and poor aortic texture, distal anastomosis bleeding is sometimes an issue [3]. At present, several techniques have been reported [1, 4–7]. The branched graft inversion technique developed by Tanaka et al. [8]. However, this technique has been only applied to elective thoracic aortic aneurysms. In this paper, we report the first application of this technique in total aortic arch replacement using the frozen elephant trunk (FET) technique for the treatment of acute type A aortic dissection and summarise our experience.

## Patients and methods

### Patients

Between January 2017 and July 2019, 109 patients (91 males) at our centre underwent total arch replacement using the FET technique for type A acute aortic dissection. The mean patient age was 47.10 ± 11.16 years. Diagnoses were confirmed by computed tomography angiography (CTA), and all patients underwent emergency surgery. The characteristics of the patients are shown in Table 1. The 4-branched graft inversion technique was applied to distal anastomosis in 57 cases (group G). Conventional end-to-end anastomosis was used in 52 cases (group C).

**Table 1:** Patient characteristics

	Total (n = 109)	Group G (graft inversion) (n = 57)	Group C (conventional) (n = 52)	*P* value
Mean age	47.10 ± 11.16	46.35 ± 12.60	47.92 ± 9.39	0.465
Male/female	91/18	49/8	42/10	0.466
Organ malperfusion				
Cerebral	3 (2.8%)	2 (3.5%)	1 (1.9%)	0.613
Coronary	6 (5.5%)	2 (3.5%)	4 (7.7%)	0.339
Kidney	7 (6.4%)	3 (5.3%)	4 (7.7%)	0.605
Spinal cord	0	0	0	
Leg	8 (7.3%)	5 (8.8%)	3 (5.8%)	0.548

All strokes were confirmed by computed tomography. Postoperative renal insufficiency was defined by the requirement for continuous renal replacement therapy. Respiratory failure was defined as more than 72 h of postoperative mechanical ventilation.

### Surgical technique

**Fig. 1:**
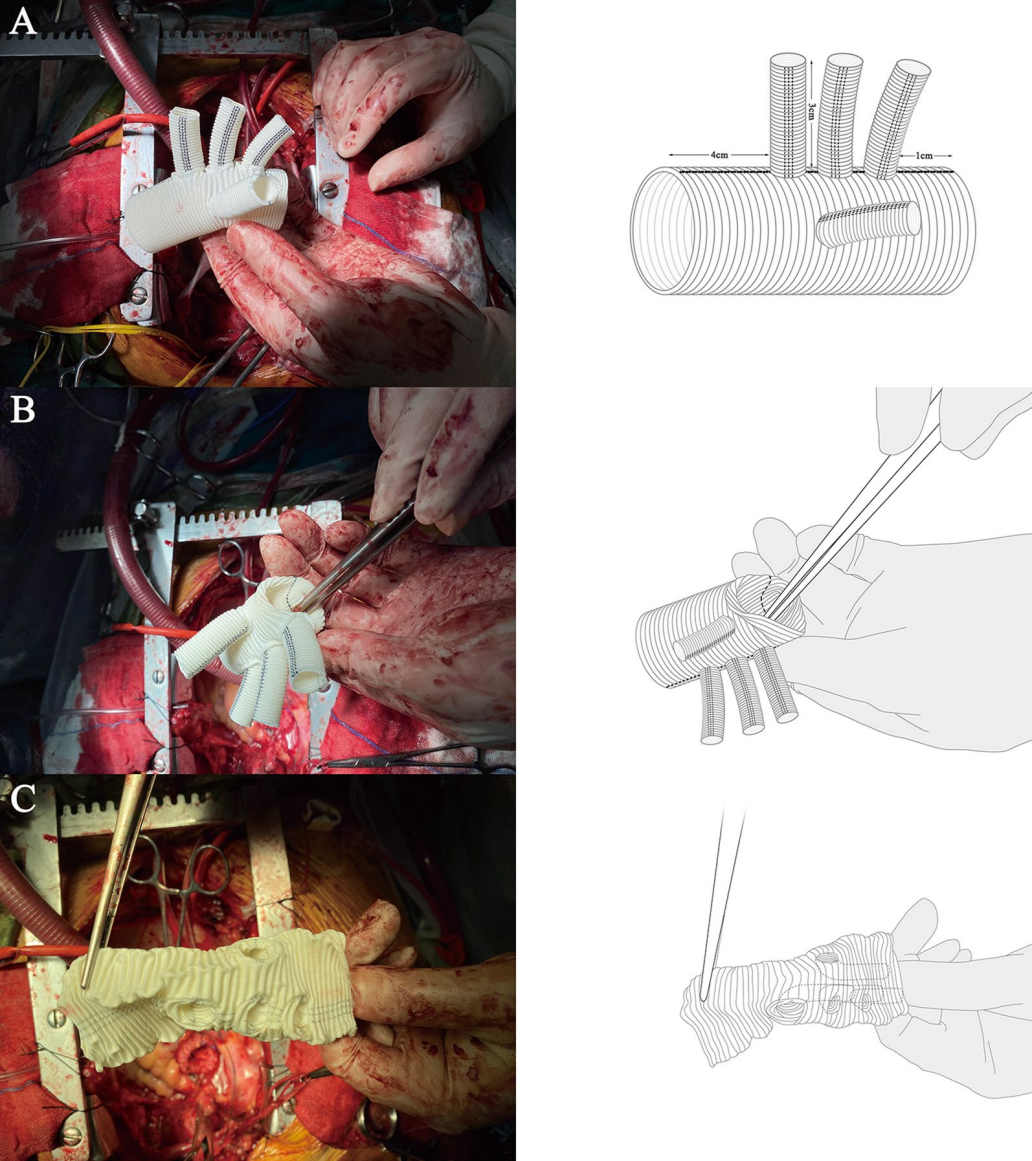
A 4-branched graft was inverted in the operation. **a** A 4-branched prosthetic graft was trimmed to the appropriate length. **b** The trimmed graft was inverted with forceps. **c** The completely inverted 4-branched graft

**Fig. 2:**
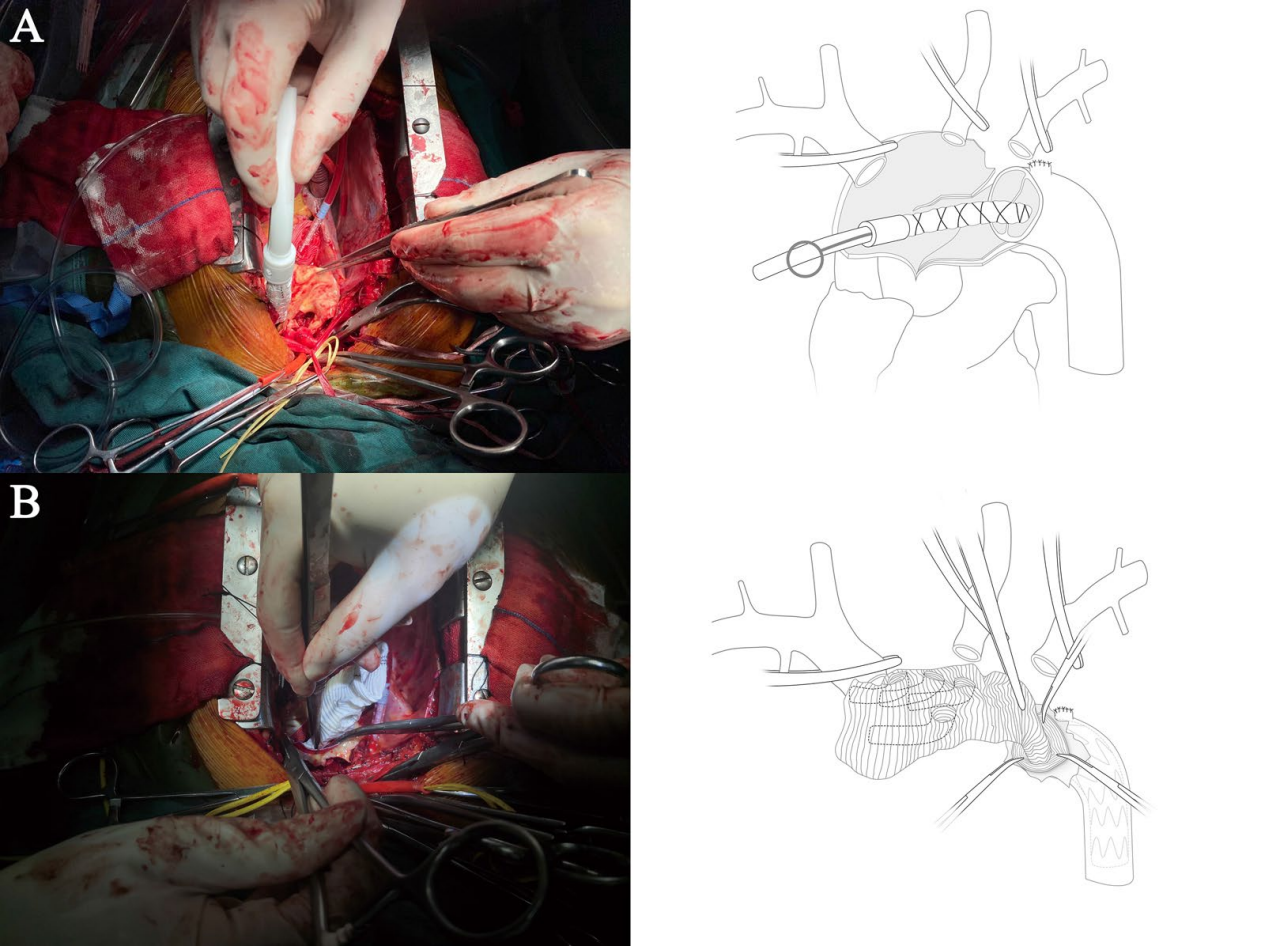
**a** A frozen elephant stent was inserted into the true lumen of the descending aorta. **b** The invaginated graft was carefully inserted into the stent graft. The openings of the 3 branches always faced the greater curvature of the aortic arch

**Fig. 3:**
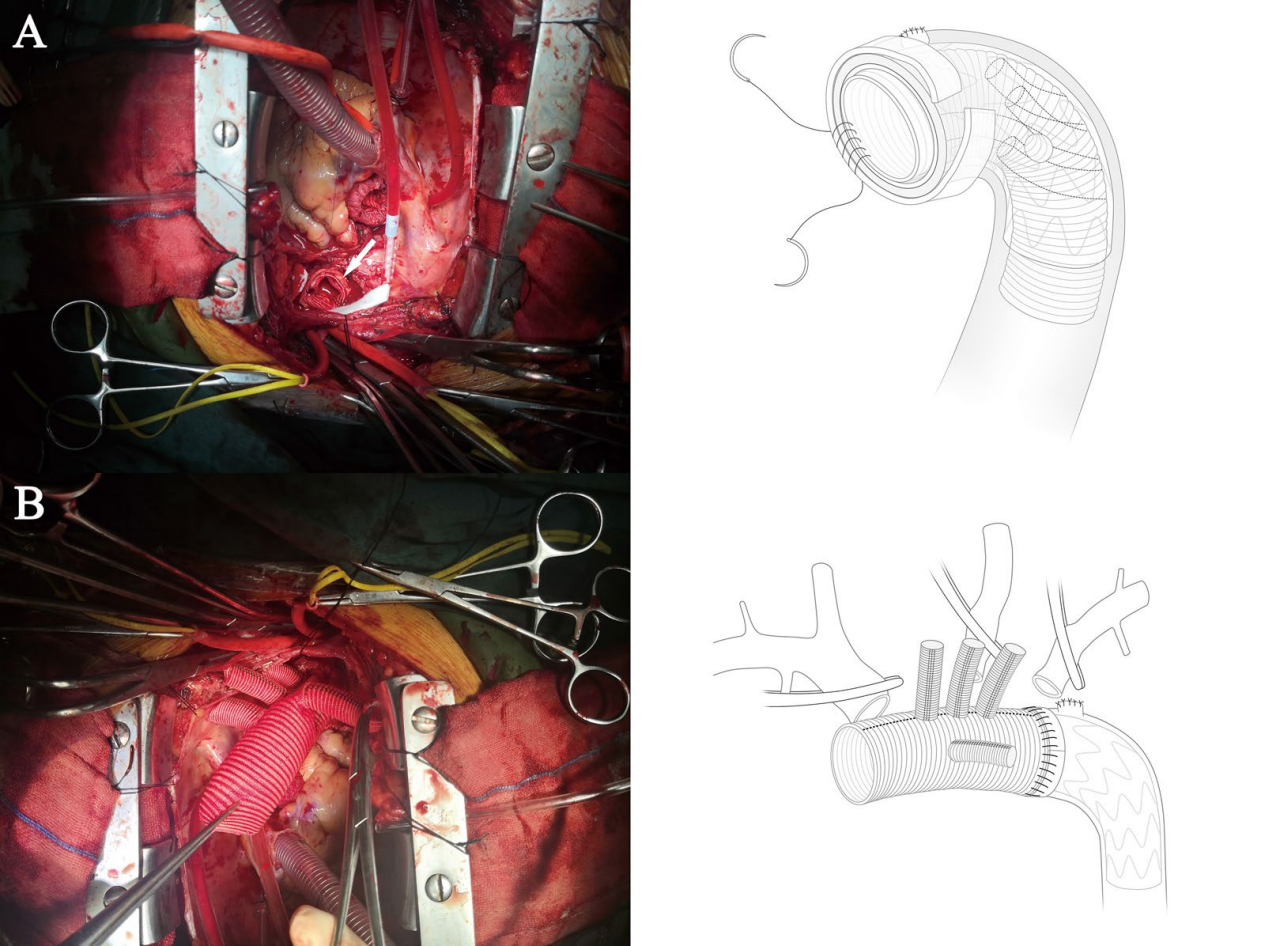
**a** The ends of the quadrifurcated graft, elephant stent, and native aortic wall were trimmed to the same level and sewn together with a Teflon felt strip using a 4–0 Prolene running suture (white arrow). **b** The 4-branched inverted graft was pulled out with forceps

**Fig. 4:**
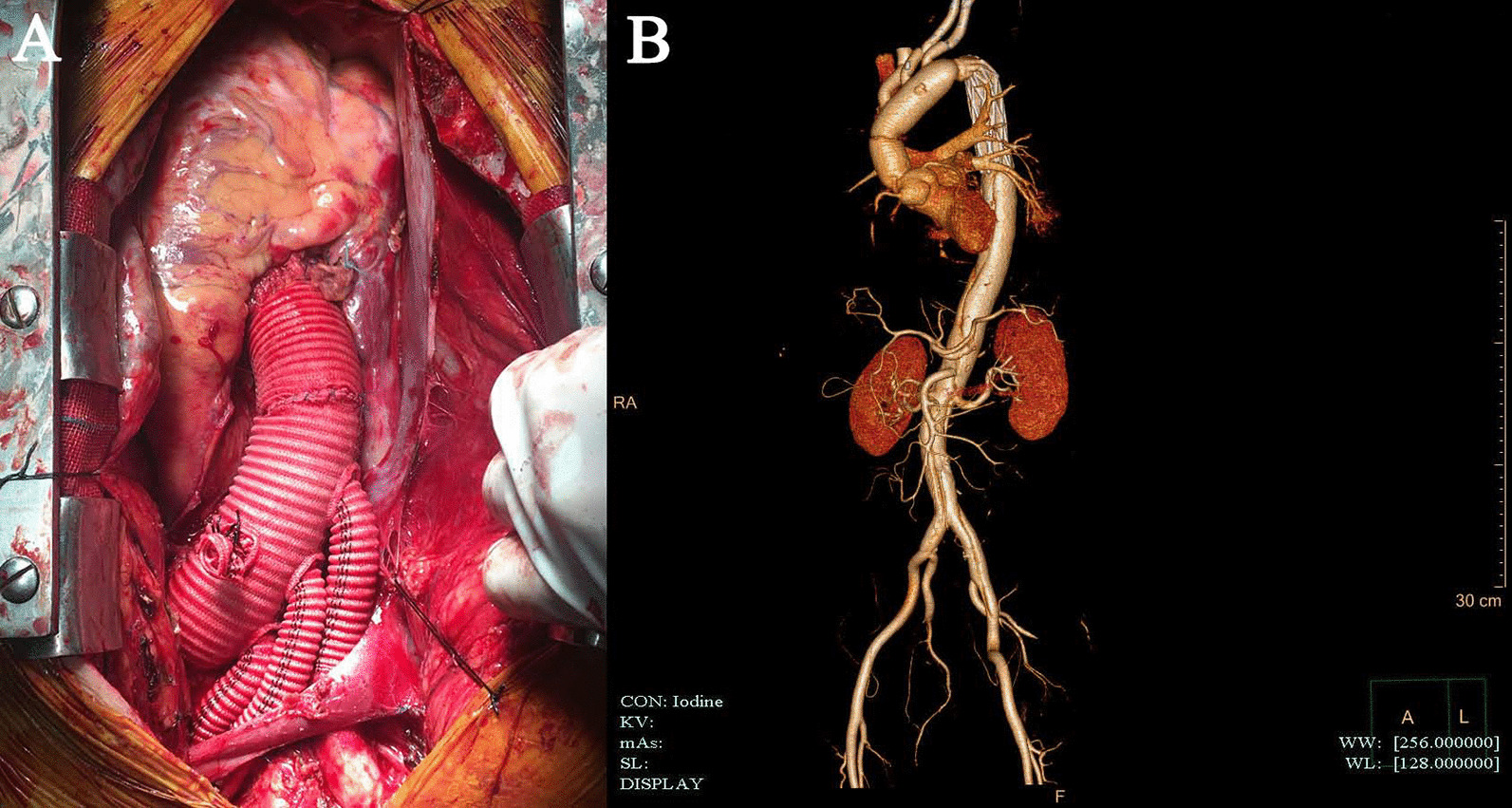
**a** Final aspect of the surgery. **b** Postoperative 3-dimensional computed tomography of the aorta

All patients underwent median sternotomy in the supine position. The right axillary artery was exposed, and an arterial cannula was inserted for cardiopulmonary bypass (CPB) and selective cerebral perfusion (SCP). A cavoatrial cannula was inserted into the right atrium. Left ventricular venting was performed through the right superior pulmonary vein. Cardiac arrest was achieved as usual. Before deep hypothermia occurred, the Bentall or aortic valve replacement procedure was performed if aortic valve lesions were present. The stepwise technique as described by Inoue et al. [9] was applied to the proximal anastomosis for patients without aortic valve lesions. Bentall, David, or aortic valve replacement was performed for patients with aortic valve lesions. Once the rectal temperature was reduced to 20 °C, deep hypothermic circulatory arrest began. The usual procedure was performed. After the proximal end of the left subclavian artery was closed by a 5–0 running suture, a frozen elephant stent (Cronus; Microport Medical Co., Ltd., Shanghai, China), which comprises two parts: a 20 mm stent-free vascular graft at the proximal end and a 100 mm self-expandable stent graft at the distal end, was inserted into the true lumen of the descending aorta (Fig. 2a).The proximal part of the stent(stent-free graft) is used to anastomose with the quadrifurcated prosthetic graft. The distal part(self-expandable stent graft) can support dissected descending aorta. In the conventional group, the distal end of a quadrifurcated prosthetic graft (Hemashield Platinum; Intervascular SAS, La Ciatot, France) was directly end-to-end anastomosed to the proximal end of the descending aorta and the frozen stent inside with Teflon felt. In group G, a 4-branched prosthetic graft was trimmed to the appropriate length (Fig. 1a) and inverted completely (Fig. 1b, c). The distal end of the graft, the 4 branches, and the proximal end of the graft are cut to about 1 cm, 3 cm, and 4 cm, respectively (Fig. 1a). Thereafter, the invaginated graft was carefully inserted into the stent graft. The openings of the three branches always faced the greater curvature of the aortic arch (Fig. 2b), which ensures the three branches are at the correct position when the graft is pulled out. The ends of the quadrifurcated graft, elephant stent, and native aortic wall were trimmed to the same level and sewed together with a Teflon felt strip using a 4–0 Prolene running suture (Fig. 3a). Finally, the 4-branched inverted graft was gently pulled out with forceps (Fig. 3b). In both groups G and C, antegrade circulatory perfusion resumed through the fourth branch of the graft. The left subclavian artery, left common carotid artery, and innominate artery were reconstructed in the order written. The 4-branched graft was anastomosed to the proximal graft at the end of the procedure (Fig. 4a).

### Statistical analysis

Data were analysed using SPSS 19 software (IBM Corp., Armonk, NY, USA) for Windows. Continuous variables are reported as means ± standard deviations (SDs). Categoric data are described as frequencies and percentages. The independent samples *t* test or chi-square test was used for comparison.

## Results

A total of 109 patients underwent ascending aorta replacement, total arch replacement, and FET implantation. The following concomitant procedures were performed: coronary artery bypass grafting (CABG) (6 patients), aortic valve replacement (3 patients), Bentall (28 patients), tricuspid valve plasty (3 patients), and subvalvular aortic stenosis resection (1 patient). The mean CPB, cardiac arrest, and circulatory arrest times were 234.95 ± 71.88 min, 168.25 ± 61.33 min, and 39.19 ± 9.45 min, respectively. The mean operative time was 366.18 ± 68.70 min. The circulatory arrest and cardiac arrest times were shorter in group G than in the conventional group (36.46 ± 7.88 min vs. 42.19 ± 10.17 min, *P* = 0.001 and 156.21 ± 55.99 vs. 181.44 ± 64.68, *P* = 0.031, respectively). No significant differences in cardiopulmonary bypass and operative times were observed between the two groups.

The hospital mortality rate was 9.2% (10/109; group G, 4/57 [7.0%]; group C, 6/52 [11.5%]). The causes of death included right heart failure (n = 2), stroke (n = 1), multiple organic failure (n = 2), sudden ventricular arrhythmia (n = 1), sepsis due to pulmonary infection (n = 2), and ischaemic organ necrosis (n = 2). Three patients (all in group C, 3/52 [5.8%]) were rechecked for bleeding. There were 7 strokes (3 [5.3%] in group G and 4 [7.7%] in group C). Additionally, 5 patients (4.6%) had paraplegia, and 4 of them recovered after treatment. Moreover, 13 patients (11.9%) required temporary continuous renal replacement therapy, and 2 patients developed the need for chronic dialysis. Respiratory failure occurred in 19 patients. Other postoperative data are shown in Table 2.

**Table 2:** Operative variables and early postoperative complications

	Total (n = 109)	Group G (graft inversion) (n = 57)	Group C (conventional) (n = 52)	*P* value
HCA time	39.19 ± 9.45	36.46 ± 7.88	42.19 ± 10.17	0.001
CA time	168.25 ± 61.33	156.21 ± 55.99	181.44 ± 64.68	0.031
CPB time	234.95 ± 71.88	231.35 ± 72.85	238.90 ± 71.30	0.586
Operation time	366.18 ± 68.70	355.70 ± 70.053	377.67 ± 65.94	0.096
Concomitant procedure				
CABG	6 (5.5%)	2 (3.5%)	4 (7.7%)	0.339
AVR	3 (2.8%)	1 (1.8%)	2 (3.8%)	0.505
Bentall	28 (25.7%)	13 (22.8%)	15 (28.8%)	0.471
TVP	3 (2.8%)	2 (3.5%)	1 (1.9%)	0.613
SAS	1 (0.9%)	0	1 (1.9%)	0.293
Re-exploration for bleeding	3 (2.8%)	0	3 (5.8%)	0.066
Hospital mortality	10 (9.2%)	4 (7.0%)	6 (11.5%)	0.414
Major complications				
Stroke	7 (6.4%)	3 (5.3%)	4 (7.7%)	0.605
Paraplegia	5 (4.6%)	2 (3.5%)	3 (5.8%)	0.573
Heart failure	6 (5.5%)	3 (5.3%)	3 (5.8%)	0.908
Respiratory failure	19 (17.4%)	11 (19.3%)	8 (15.4%)	0.591
Renal insufficiency (CRRT)	13 (11.9%)	5 (14.0%)	8 (9.6%)	0.287

*HCA* hypothermic circulatory arrest, *CA* cardiac arrest, *CPB* cardiopulmonary bypass, *CABG* coronary artery bypass grafting, *AVR* aortic valve replacement, *TVP* tricuspid valve plasty, *SAS* subvalvular aortic stenosis, *CRRT* continuous renal replacement therapy

## Discussion

The FET technique is an effective approach with good long-term results for chronic or acute aortic dissection involving the arch [10–12]. In conventional distal anastomosis, the quadrifurcated prosthetic graft does not fit well with the stent graft and the native aortic wall [7]. However, the anastomosis is relatively quick owing to the limited circulation arrest time. Bleeding at the distal anastomosis is a tricky problem. The reverse stepwise technique is a good method [13], but it involves the connection of a graft between the 4-branched graft and frozen stent, which increases the risk of bleeding at the graft-to-graft anastomosis. Most importantly, the hypothermic circulatory time increases [1]. The branched graft inversion technique was first developed by Tanaka et al. It is used for aortic arch replacement in elective surgery for thoracic aortic aneurysms without the frozen elephant stent procedure. Tanaka also mentioned the limitation of this technique: it is not suitable for acute aortic dissection because of the presence of dissection, which can cause an intima tear [8]. We first applied the 4-branched graft inversion technique during the FET procedure for the treatment of acute type A aortic dissection. During the process of prosthetic graft insertion, the aortic intima is isolated and unwounded because of the frozen stent in the descending aorta. The deep hypothermic circulatory arrest and cardiac arrest times are greatly reduced using this technique. In 57 patients, uncontrolled distal anastomotic bleeding did not occur, and statistical analysis indicated that the rates of postoperative stroke, other complications, and mortality were the same as those who had undergone the traditional anastomosis method.

This method has several advantages. First, the quadrifurcated prosthetic graft is directly anastomosed with the frozen stent, which decreases the hypothermic circulatory arrest time. Second, the quadrifurcated prosthetic graft fits to the stent graft and native aortic wall well, speeding up the anastomosis. Third, uncontrollable bleeding almost never occurs. Fourth, a good surgical view allows rapid anastomosis without interruption by the 4-branched graft.

When using this method, it is also necessary to pay attention to various factors. First, the 4-branched prosthetic graft must be trimmed to the appropriate length. In our centre, we cut it as short as possible based on the premise that this will guarantee anastomosis. The distal end of the graft, the 4 branches, and the proximal end of the graft are cut to about 1 cm, 3 cm, and 4 cm, respectively. Second, during the insertion process, the openings of the 3 branches should always face the greater curvature of the aortic arch.

## Conclusions

For total aortic arch replacement with the FET procedure, the 4-branched graft inversion technique involves simple and fast distal anastomosis for acute type A aortic dissection. It provides effective and confirmed haemostasis and reduces the circulatory arrest time.

## Data Availability

Please contact author for data requests.

## Abbreviations

FET: Frozen elephant trunk
CPB: Cardiopulmonary bypass
CTA: Computed tomography angiography
SCP: Selective cerebral perfusion
CABG: Coronary artery bypass grafting
